# PRRG4 function reveals that Robo trafficking is evolutionarily conserved

**DOI:** 10.1371/journal.pgen.1006927

**Published:** 2017-08-31

**Authors:** Jimena Berni

**Affiliations:** Department of Zoology, University of Cambridge, Cambridge, United Kingdom; New York University, UNITED STATES

Achieving a correct set of neuronal connections during development is key for a healthy functioning nervous system. Autism, which is characterised by impairments in social interaction, language, and range of interests, has been hypothesised to originate from defective synaptic function and abnormal brain connectivity [1,2]. Moreover, genetic alterations such as the deficiency in proline-rich carboxyglutamic acid protein 4 (PRRG4) have been associated with autistic features present in WAGR syndrome (Wilm’s tumour, aniridia, genitourinary anomalies and “mental retardation”). Therefore, understanding the genetic mechanisms underlying the assembly of brain circuits is likely to be essential for the design of new diagnostic tools and therapeutic strategies for Autistic Spectrum Disorders (ASD). In this issue of *PLOS Genetics*, Justice et al. link genetic alterations and neural circuitry development, revealing a novel role for the PRRG4 as a regulator of Roundabout (Robo) receptor subcellular localization in the nervous system [3].

**Fig 1:**
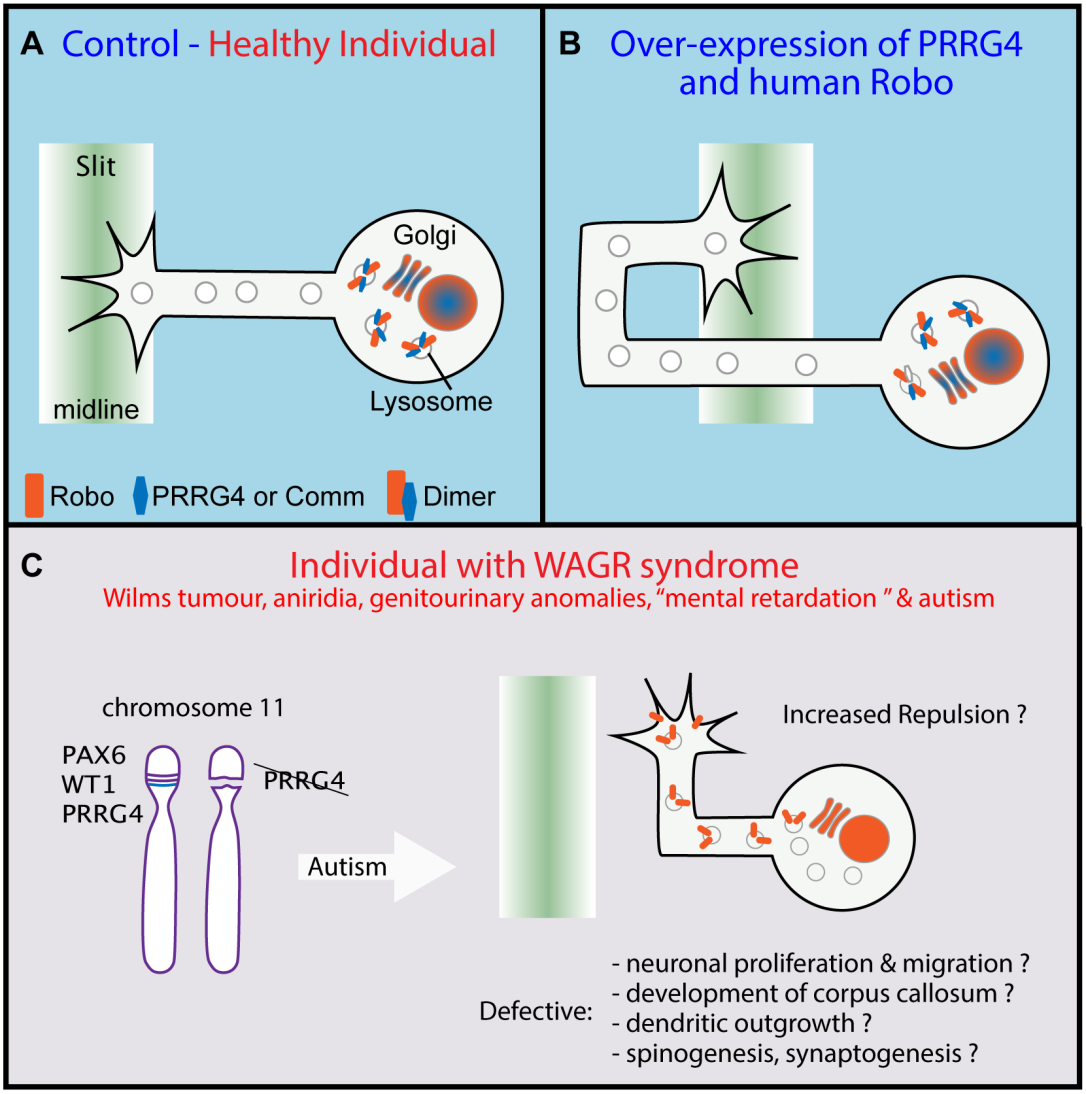
Proline-rich carboxyglutamic acid protein 4 (PRRG4) is a Commissureless (Comm) homologue. (A) In *Drosophila* control, before and during midline crossing, Comm dimerises with Robo and induces its degradation, preventing its trafficking to the axon growth cone extracellular membrane. A similar situation is suggested (red text) in vertebrates but relies on PRRG4-Robo dimers. (B) Overexpression of PRRG4 and human Robo induces a *Drosophila* Robo null phenotype with axons crossing aberrantly the midline indicating that PRRG4 acts as a Comm homologue. (C) PRRG4 deletion is associated with Autistic Spectrum Disorder (ASD) in WAGR syndrome. The results allow the hypothesis that Robo trafficking deregulation may underlie ASD through multiple mechanisms. *Adapted from* [6] *and* [3].

Both in vertebrates and invertebrates, the Slit/Robo repulsive pathway plays a major role in regulating how commissural neurons cross the midline while they navigate towards their targets on the contralateral side of the nervous system (reviewed by [4]). As the neuron extends towards and across the midline, the Robo receptor is absent from the growth cone of commissural axons, desensitizing it from the repulsive Slit signal emanating from the midline [5]. Robo extracellular membrane levels increase after the growing axon crosses the midline, guaranteeing that it will neither stall at the midline or cross back. In *Drosophila*, the decrease in Robo in the growth cone membrane of precrossing commissural neurons is regulated by Commissureless (Comm), which sorts Robo to the late endosome for degradation [6,7] (Fig 1). In the vertebrate hindbrain, the protein Rig1/Robo3 has been shown to regulate the responsiveness to Slit but through a different mechanism, probably dimerising with Robo1 and rendering it irresponsive to Slit [8]. So far, however, the way Robo is regulated in the vertebrate forebrain is unknown and it is tantalizing to think that a functional Comm homologue could be performing this function.

Justice et al. present their finding of a vertebrate functional homologue of Comm capable of regulating midline crossing in the fruit fly heterologous system. Because there are no gene sequence homologues of Comm in vertebrates, they decided to guide their search looking for the protein motif that could determine conserved function. There is little sequence requirement for Comm sorting function [6,9] and the authors embarked on an uncertain but ambitious quest focusing on an extended version of the L/PPxY motif. This strategy proved successful and they found several candidates in different species. They then used the reverse strategy and looked for a putative motif from the candidate proteins that could be found in Comm. The PRRG family of proteins presented the higher homology containing an N-terminal Gla domain that seems degenerated in Comm and the LPxY motif. To test *in vivo* the functional role of the candidate proteins, they overexpressed them in the nervous system of *Drosophila*. A functional homologue of Comm is expected to induce a Robo null phenotype that manifests itself by increased midline crossing as the consequence of decreased receptor availability [10]. PRRG4 induces midline crossing defects, the penetrance of which was magnified when overexpressed with human Robo (Fig 1). Finally, to understand the mechanism of action of PRRG4, Justice et al. overexpressed murine and *Drosophila* proteins in cell culture and demonstrated that PRRG4 species specifically recruits Robo to the endoplasmic reticulum/Golgi and that it induces a decrease in protein levels, probably through targeted degradation. This shared function between Comm and PRRG4 implies that the mechanism of Robo trafficking is evolutionarily conserved and that Comm/PRRG proteins may belong to an ancient family of cell surface protein effectors.

Interestingly, haploinsufficiencies of PRRG4 or Pax 6 are candidate genes underlying ASD in patients with WAGR syndrome. The finding of a likely role of PRRG4 in regulating Robo trafficking in vertebrates suggests that defects in the Slit/Robo pathway may underlie the ASD features. Considering that Slit/Robo signalling pathway is involved in several aspects of forebrain development (e.g., neuronal proliferation and migration, development of corpus callosum, dendritic outgrowth, and spinogenesis [11]), the discovery presented by Justice et al. represents a unique opportunity to further investigate the complexity of the Robo-dependent signalling pathway for the development of the vertebrate brain in health and disease.
